# Dual mode OPV-OLED device with photovoltaic and light-emitting functionalities

**DOI:** 10.1038/s41598-018-29806-8

**Published:** 2018-07-31

**Authors:** Takayuki Chiba, Daichi Kumagai, Kazuo Udagawa, Yuichiro Watanabe, Junji Kido

**Affiliations:** Graduate School of Organic Materials Science, Yamagata University, 4-3-16 Jonan, Yonezawa, Yamagata, 992-8510 Japan

## Abstract

The rapid development of organic optoelectronic devices such as organic photovoltaics (OPVs) and organic light-emitting devices (OLEDs) is largely attributable to their advantageous properties of their large area, ultrathin thickness, flexiblility, transparency, and solution processability. Herein, we fabricate and characterize a dual mode OPV-OLED device with three-terminal structure comprising a polymer-based bulk-heterojunction inverted OPV unit and a top-emission white phosphorescent OLED unit back-to-back connected via intermediate metal alloy electrode. Sputter-deposited indium tin oxide was used as a transparent cathode of the inverted OPV unit, whereas Ag-doped Al served as a common OPV/OLED anode, allowing the decoupling of electricity generation and light mission functions. Notably, the doping of Al by Ag facilitated the reduction of surface roughness, allowing the above electrode to be used as a common anode and dramatically reducing the leakage current. Finally, the top-emission OLED unit featured an ultrathin layer of Ag-doped Mg as a semitransparent cathode. Thus, successful integration of the OPV-OLED elements results in the decoupling of electricity generation and light emission functionalities, achieving a power conversion efficiency of 3.4% and an external quantum efficiency of 9.9%.

## Introduction

Organic optoelectronic devices such as organic photovoltaics (OPVs)^1–3^ and organic light-emitting devices (OLEDs)^4–6^ have attracted considerable attention due to exhibiting the advantages of large area, ultrathin, flexibility, transparency, and solution processability. Specifically, the active layer of bulk-heterojunction OPVs typically consisted of electron-donor (p-type) and electron-acceptor (n-type) semiconductors^7,8^, with polymer-based single bulk-heterojunction OPVs achieving power conversion efficiencies (PCEs) of up to 12%^9–14^. Tandem OPVs, i.e., those with multi-junction structures, have been demonstrated to effectively capture solar light, converting a rarely utilized part of the solar spectrum with the help of complementally-absorption polymers and thus exhibiting reduced energy loss^15–19^. Therefore, semitransparent ultrathin Ag layer, metal oxides, conductive polymers were used as the intermediate layer of tandem OPVs. Similarly, the development of OLEDs has been fueled by their potential used in energy-saving flat light sources and display applications. Recently, the design of novel materials and device structures has allowed the external quantum efficiencies (EQEs) of OLEDs to exceed 30%^20–24^. In tandem OLEDs, developed to simultaneously achieve high efficiency and long operational lifetime, multiple light-emitting units are connected in series via a charge generation layer (CGL)^25–28^. Additionally, top-emission OLEDs, in which the conventional reflective metal electrode is replaced by a semitransparent ultrathin one, are widely used to improve the aperture ratio of OLED displays^29–31^.

Recently, the integration of OPVs and OLEDs in a series-connected directory has led to development of new functional devices, e.g., those used for light upconversion and infrared sensing device^32–35^. In such devices, the OPV unit acts as a CGL under light irradiation, with the photogenerated carriers being subsequently injected into the OLED unit. Light upconversion devices convert near-infrared radiation into visible light, thus being of high importance for h night vision applications^33^. On the other hand, the use of semitransparent metal electrodes for OPV–OLED integration was reported to result in high-color-purity of electroluminescence (EL)^36,37^, with the OPV unit comprising a mixture of zinc phthalocyanine (ZnPc) and fullerene (C_60_), whereas the OLED emissive layer contained tris(8-hydroxyquinoline)aluminum (Alq_3_) and 10-(2-benzo-thiazolyl)-1,1,7,7-tetramethyl-2,3,6,7-tetrahydro-1H,5H,11H-[l]1benzopyrano[6,7,8-ij]-quinolizin-11-one (C545T). The peak EL wavelength of the Alq_3_:C545T–based OLED unit was obtained as 530 nm, whereas the transparency of the ZnPc:C_60_–based OPV unit was less than 70% at 530 nm, which implies that OLED-emitted light unit was able to pass through the OPV unit with a small absorption loss. However, in this integrated device, light incident into the OPV was required to be oriented in the same direction as that emitted by the OLED. Therefore, the development of a dual mode OPV-OLED device is required to decouple the above directions without absorption loss.

In addition, the dual mode OPV-OLED device enables simultaneously photovoltaic and light-emitting characteristics in one device. These novel features of dual mode device can be applied to the “smart window blinds”. In general, window blinds block out the sunlight, whereas smart window blinds by dual mode OPV-OLED device harness the potential sunlight as a solar power during the day. Moreover, dual mode OPV-OLED device can be used for the lighting application at night.

Herein, to decoupled electricity generation and light emission function, we fabricated a dual mode OPV-OLED device with three terminal structure (active are = 1 cm^2^) featuring a polymer-based bulk-heterojunction inverted OPV unit and a top-emission white phosphorescent OLED unit back-to-back connected via an intermediate metal alloy electrode Sputter-deposited indium tin oxide (ITO) with relatively high transmittance (up to 90%) was used as the transparent cathode of the inverted OPV unit. The intermediate connecting electrode comprising Ag-doped Al and played a key role in realizing the individual operation of OPV and OLED units, with its smooth surface^38,39^ resulting in an acceptably small device leakage current. Ultrathin Ag-doped Mg was used as a semitransparent cathode for the top-emission OLED unit, and the fabricated dual mode device exhibited a PCE of 3.4% and an EQE of 9.9%.

## Results and Discussions

**Figure 1:**
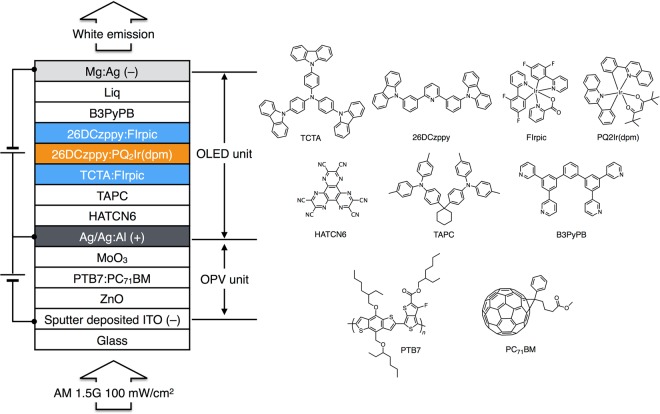
Structures of the dual mode OPV-OLED and its chemical constituents.

To realize the abovementioned dual mode device, we employed a three-terminal structure, namely ITO transparent cathode/inverted OPV/Ag-doped Al intermediate connecting anode/top-emission OLED/Ag-doped Mg semitransparent cathode, as shown in Fig. 1. The active area of fabricated device equaled 1 cm^2^, being fairly large compared to the conventional laboratory-scale value of 0.04 cm^2^. Thus, the OPV unit was irradiated by solar light from the transparent ITO–coated glass side, whereas OLED–emitted light passed through the semitransparent ultrathin Ag-doped Mg cathode. We prepared the various deposition masks with active area of 1 cm^2^ such as sputter-deposited ITO mask, intermediate electrode mask, OLED mask, and transparent top electrode mask, as shown in Fig. S1.

The polymer-based bulk-heterojunction inverted OPV unit of the dual mode device utilized *p*-type poly[(4,8-bis-(2-ethylhexyloxy)-benzo(1,2-b:4,5-b′)dithiophene)-2,6-diyl-alt-(4-(2-ethylhexyl)-3-fluorothieno[3,4-b]-thiophene-)-2-carboxylate-2–6-diyl]] (PTB7) and *n*-type [6,6]-phenyl-C_71_-butyric acid methyl ester (PC_71_BM) as an active layer materials. ZnO nanoparticles were used as an electron-collecting layer, and MoO_3_ was used as a hole-collecting layer. The inverted OPV unit had the following configuration: sputter-deposited ITO (130 nm)/ZnO (30 nm)/PTB7:PC_71_BM (100 nm)/MoO_3_ (8 nm)/Ag (30 nm), with the corresponding energy diagrams shown in Fig. S2. To prepare sputter-deposited ITO with an active area of 1 cm^2^ and simultaneously achieve high transmittance and low sheet resistance, we investigated the effect of annealing during ITO deposition. Thus, ITO sputter-deposited without annealing showed a high sheet resistance of 56 Ω square^−1^ at a thickness of 130 nm, whereas deposition accompanied by annealing at 200 °C resulted in decreased resistance (28 Ω square^−1^ at thickness of 130 nm). The transmittance (450–620 nm) of sputter-deposited ITO film annealed at 200 °C exceeded 90%, being almost identical to the value obtained for the ITO film sputter-deposited without annealing (Fig. S3). However, both annealed and non-annealed ITO films showed low transmittance in the range of 300–400 nm. The obtained root-mean-square (*R*_*a*_) and maximum (*R*_*max*_) heights of annealed and non-annealed ITO films revealed that the latter (*R*_a_ = 0.68 nm, *R*_max_ = 8.28 nm) was rougher than former (*R*_a_ = 0.33 nm, *R*_max_ = 6.71 nm) (Fig. S4). ZnO nanoparticles dispersed in 2-ethoxyethanol were spin-coated onto the sputter deposited ITO film, being subsequently overcoated by PTB7:PC_71_BM (2:3 w/w, solution in chlorobenzene:1,8-diiodooctane = 97:3 v/v), which showed broadband absorption at 300–750 nm (Fig. S5).

**Figure 2:**
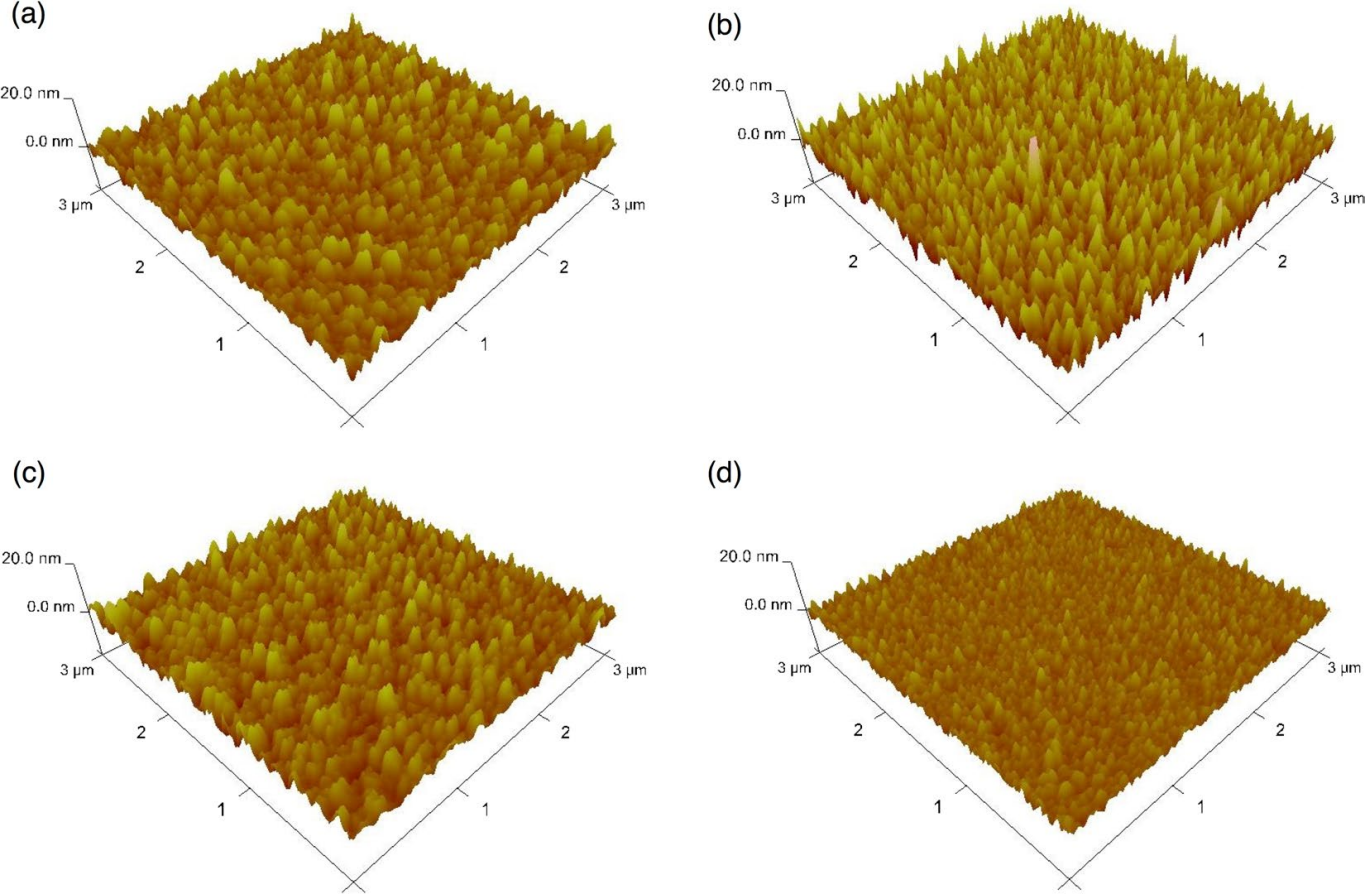
AFM images of (**a**) Ag film, (**b**) Al on Ag film, (**c**) 30 wt% Ag-doped Al on Ag film, and (**d**) 60 wt% Ag-doped Al on Ag film.

**Figure 3:**
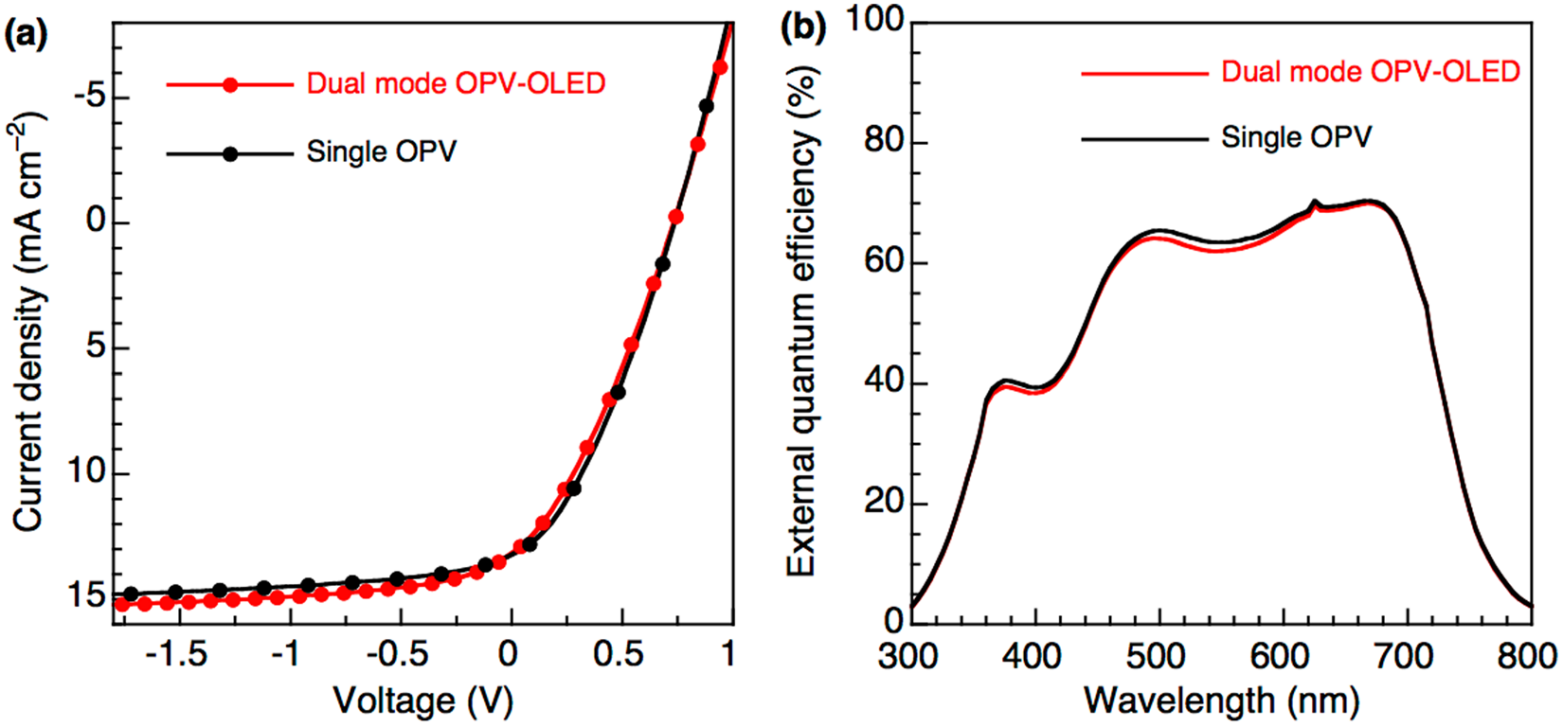
(**a**) *J-V* curves and (**b**) *EQE* spectra of the OPV unit in the dual mode device and those of single inverted OPV.

The intermediate electrode was utilized for charge collection from the OPV unit and charge injection into the OLED unit, with its smooth surface being of high importance. Ag films are commonly used as inverted OPV anodes due to featuring appropriate energy level alignment. However, the Ag film employed herein showed a high surface roughness (*R*_*a*_ = 1.84 nm, and *R*_*max*_ = 18.3 nm) **(**Fig. 2a)), which resulted in increased leakage current when OLED unit was directly fabricated onto high roughness Ag film. To reduce surface roughness, we fabricated an Ag-doped Al alloy electrode by co-evaporation of Ag and Al. Figure 2b–d show atomic force microscopy (AFM) images of Ag-doped (Ag contents of 0, 30, and 60 wt%, respectively) Al films onto Ag film. The surface roughness of the Ag-doped Al film with a thickness of 70 nm decreased from 2.71 to 1.16 nm (*R*_*a*_) and from 36.4 to 10.7 nm (*R*_*max*_) as the Ag content increased from 0 to 60 wt%, reflecting the fact that co-evaporation prevented the aggregation of homogeneous metal clusters and hence reduced the device leakage current and optical loss of the device^38^. In addition, relatively small *R*_*a*_ and *R*_*max*_ values (2.13 and 21.2 nm, respectively) were observed even when Ag-doped Al was deposited over the whole inverted OPV unit (Fig. S6). Figure 3a shows the current density–voltage curves for the OPV in the dual mode device and for a single inverted OPV under typical air mass 1.5 global irradiation (AM1.5 G, 100 mW cm^−2^). The dual mode OPV-OLED featured PCE = 3.31%, short-circuit current (*J*sc) = 13.2 mA cm^−2^, open-circuit voltage (*V*oc) = 0.73 V, and fill factor (FF) = 33%, with the observed efficiency thus being almost identical to that of the single inverted OPV (PCE = 3.36%, FF = 35%). This result suggested that the OPV unit of dual mode device could be effectively operated as an electricity generation source similarly to a conventional single OPV. Figure 3b shows the external quantum efficiency (EQE) curves the above OPVs, revealing their close similarity. Notably, a low EQE was observed at 300–450 nm, which was attributed to the low transmittance of sputter-deposited ITO film in this short-wavelength range. The characteristics of the utilized OPVs are summarized in Table 1.

**Table 1:** OPV characteristics of composite and single devices.

Device	*J*_*sc*_ (mA cm^−2^)	*V*_*oc*_ (V)	FF	PCE (%)
Dual mode OPV-OLED	13.17	0.73	0.33	3.13
Single OPV	13.22	0.73	0.35	3.36

**Figure 4:**
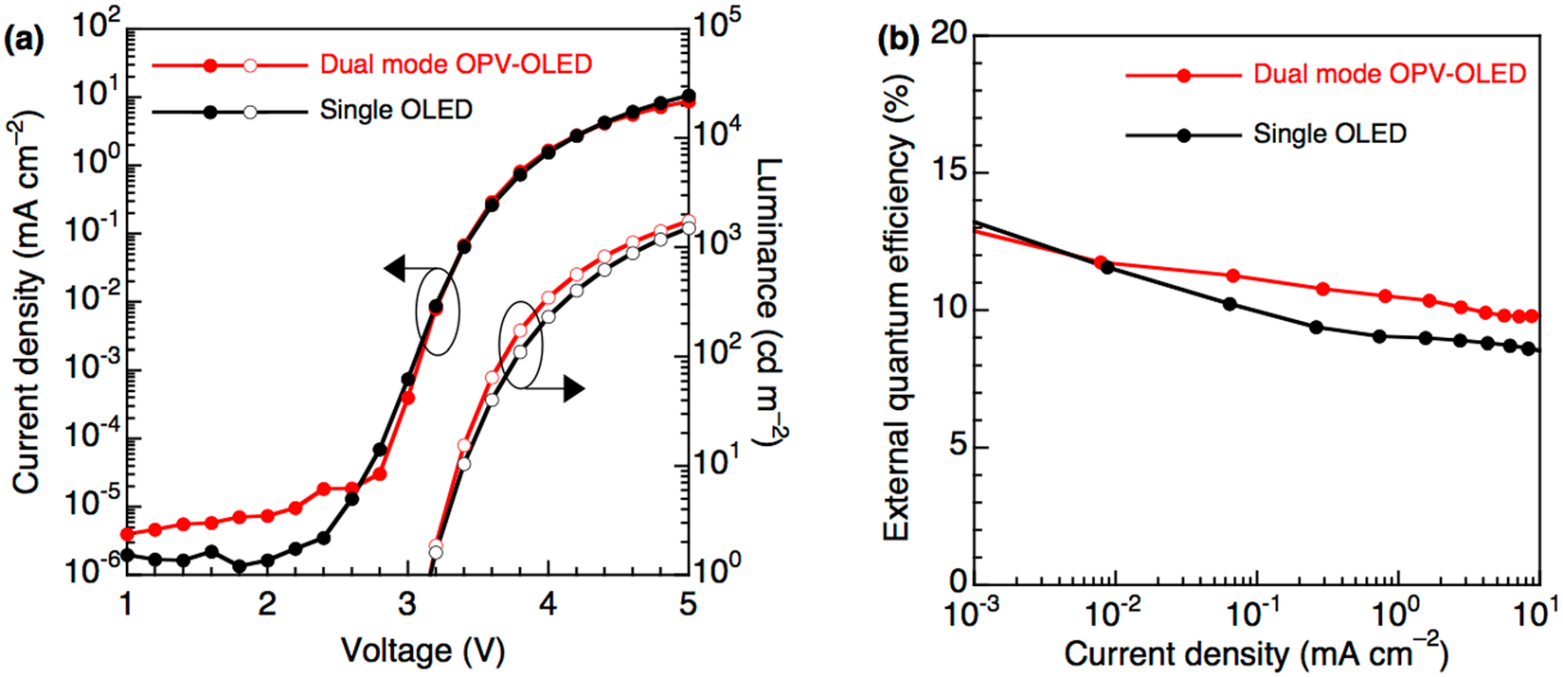
(**a**) *J–L-V* and (**b**) *EQE–J* curves of dual mode OPV-OLED and a single OLED.

The top-emission white OLED unit was fabricated onto the 60 wt% Ag-doped Al intermediate connecting anode using a blue phosphorescent emitter, bis[2-(4,6-difluorophenyl)pyridinato-*C*^2^,*N*](picolinato)iridium(III) (FIrpic), and an orange phosphorescent emitter, iridium(III) bis-(2-phenylquinoly-N,C2′)dipivaloylmethane (PQ2Ir(dpm)). Two host materials were used in the emissive layer, namely 4,4′,4′′-tris(carbazol-9-yl)-triphenylamine (TCTA) as a hole-transporting material, and 2,6-bis(3-(9-carbazol-9-yl)phenyl)pyridine (26DCzPPy) as a bipolar materials. The orange emissive layer, 26DCzPPy:PQ2Ir(dpm), was inserted between two blue emissive layers (TCTA:FIrpic and 26DCzPPy:FIrpic) to obtain both blue and orange emission^40^. 1,4,5,8,9,11-hexaazatriphenylene hexacarbonitrile (HATCN6), am organic electron-acceptor, was used for hole injection layer from the metal anode, and 1,1-bis-(4-bis(4-tolyl)-aminophenyl)cyclohexene (TAPC) was used as a hole-transport layer. 3,3′′,5,5′′-tetra(3-pyridyl)-1,1′;3′,1′′-terphenyl (B3PyPB) and (8-quinolinolato)lithium (Liq) were used as electron-transport and electron-injection layers, respectively. The triplet energy levels of TAPC (2.95 eV) and B3PyPB (2.69 eV) were located above those of the phosphorescent emitters FIrpic (2.62 eV) and PQ_2_Ir(dpm) (2.10 eV), which prevented the triplet exciton quenching of phosphorescent emitters at the interface between the emissive layer and adjacent charge transport layers. The top-emission white OLED unit configured as reflecting Ag:Al anode (100 nm)/HATCN6 (5 nm)/TAPC (65 nm)/TCTA:10 wt% FIrpic (5 nm)/26DCzPPy:5 wt%PQ_2_Ir(dpm) (2 nm)/26DCzPPy:10 wt%FIrpic (5 nm)/B3PyPB (55 nm)/Liq (2 nm)/semitransparent Mg:Ag cathode (15 nm), with the corresponding energy diagram shown in Fig. S7. The EL spectra of the top-emission OLED unit of dual mode device corresponded to white emission from both phosphorescence emitters (FIrpic and PQ_2_Ir(dpm)), (Fig. S8), with the corresponding current density–luminance–voltage *(J*–*L–V*) curves shown in Fig. 4a. Notably, the dual mode device featured *J-V* curves very similar to those of the single top-emission OLED with an active area of 1 cm^2^, and no leakage current was observed due to the reduced surface roughness of the intermediate Ag-doped Al electrode. On the other hand, a high leakage current was observed when the intermediate electrode comprised non-doped Al as shown in Fig. S9, which was attributed to the high surface roughness of the pure Al film. The surface roughness of these anodes was dominant factor in not only dual mode devices, but also single OLEDs. The leakage current of single OLED was dramatically reduced by using the Ag-doped Al electrode compared to the non-doped Al electrode one, as shown in Fig. S10. Therefore, the smooth Ag-doped Al electrode could be used to effectively operate the top-emission OLED unit in dual mode device. At luminance of 1, 100, and 1000 cd m^2^, the driving voltages of the above device equaled 3.10, 3.67, and 4.52 V at, respectively, which indicated that the holes and electrons were effectively injected into the top-emission OLED unit from both the Ag-doped Al anode and the Ag-doped Mg cathode. Moreover, the above unit exhibited a power efficiency (PE) of 13.8 lm W^−1^ and an EQE of 9.9% at 1000 cd m^−2^, respectively, thus featuring a performances similar to that of the single top-emission OLED (Fig. 4b). The characteristics of the utilized OLEDs are summarized in Table 2. Thus, based on the obtained results, the dual mode OPV-OLED device could be effectively operated as both a top-emission white OLED and a single-OLED.

**Table 2:** OLED characteristics of composite and single devices.

Device	V_on_ (V)	V (V)	PE (lm W^−1^)	CE (cd A^−1^)	EQE (%)
Dual mode OPV-OLED	3.10	4.52	13.8	19.9	9.9
Single OLED	3.11	4.67	9.6	14.3	8.6

## Conclusion

In summary, we have successfully fabricated and characterized a dual mode three-terminal OPV-OLED device comprising a polymer-based bulk-heterojunction inverted-OPV unit and a top-emission white phosphorescent OLED unit back-to-back connected by an intermediate metal alloy electrode. Sputter-deposited transparent ITO was used as the cathode of the inverted OPV unit, whereas the abovementioned metal alloy (Ag-doped Al) electrode was used to enable the decoupling of electricity generation and light emitting functions. The doping of Al by Ag played a significant role in the reduction of surface roughness, resulting in the almost complete absence of leakage current. Ultrathin Ag-doped Mg was used as a semitransparent cathode for the top-emission white OLED unit of dual mode device, which exhibited a PCE of 3.4% and an EQE of 9.9%.

## Methods

### Materials

PTB7 and PC_71_BM were purchased from Solarmer Energy. HATCN6, TAPC, TCTA, and Liq were purchased from eRay. 26DCzPPy and FIrpic were purchased from Chemipro Kasei, and PQ_2_Ir(dpm) was purchased from Lumtec. ZnO and B3PyPB were synthesized according to the kown literature procedure.

### ITO film fabrication

Glass substrates were cleaned with ultra-purified water and neutral detergent, being subsequently, dry-cleaned by 10-min exposure to an UV-ozone ambient. The cleaned substrates were coated with ITO films at room temperature by the radio frequency (RF) sputtering (NRF-technologies NR05NP-03) using an ITO target (90% In_2_O_3_–10%SnO_2_, 99.99%) supplied by Kojundo Chemical. The target–substrate distance equaled 200 mm, and the sputtering chamber was evacuated to less than 5 × 10^−5^ Pa prior to deposition. High-purity Ar (99.999%) and O_2_ (99.999%) were introduced at rates of 40 and 0.4 sccm, respectively. Before deposition, the target was decontaminated by 5-min pre-sputtering in Ar-O_2_. The working pressure equaled 0.3 Pa, and the RF power was set to 160 W, resulting in the deposition of a 130-nm-thick ITO film with an active area of 1 cm^2^.

### Device fabrication

To fabricate an inverted OPV with a polymer-based bulk-heterojunction, a dispersion of ZnO nanoparticles (10 mg mL^−1^) in 2-ethoxyethanol was spin-coated onto a cleaned ITO substrate and annealed at 100 °C for 10 min to produce a 30-nm-thick layer. A blend of PTB7:PC_71_BM (2:3 w/w) in chlorobenzene:1,8-diiodooctane (97:3 v/v) with a concentration of 25 mg mL^−1^ was spin coated onto ZnO to afford a 100-nm-thick layer. All spin coating and annealing procedures were performed in a nitrogen-filled glove box (<0.1ppm O_2_ and H_2_O). Other layers (MoO_3_, Ag, and Ag-doped Al) were deposited by thermal evaporation in vacuum (~10^−5^ Pa). To fabricate the top-emission white phosphorescent OLED unit, functional organic layers were deposited onto the Ag-doped Al anode by thermal evaporation in vacuum (~10^−5^ Pa) using an organic patterned shadow mask. The top cathode (Ag-doped Mg) was deposited by co-evaporation (Ag:Mg 1:9), and was patterned using a shadow mask with an array of 1-cm^2^ openings without breaking the vacuum (~10^−5^ Pa). Immediately after preparation, the obtained device was encapsulated under a nitrogen atmosphere using epoxy glue and transparent glass lids.

### Characterization

Film thickness was measured using Dektek8 profile meter, and the sheet resistivity of ITO film was measured utilizing a conventional four-probe technique. Surface roughness was analyzed using a Bruker Dimension Icon atomic force microscope. The optical transmittance of ITO film was measured using a Shimadzu UV-3150UV–vis–NIR spectrophotometer. To characterize the bulk-heterojunction inverted OPV, current density–voltage curves were recorded using a Keithley 2400 source measure unit. Light intensity was determined by a monosilicon detector (with a KG-5 visible color filter) calibrated by the National Renewable Energy Laboratory to reduce spectral mismatch. For the characterization of the top-emission white phosphorescent OLED unit, EL spectra were acquired using an optical multichannel analyzer (Hamamatsu Photonics PMA-11). Current density–voltage and luminance–voltage curves were recorded using a Keithley source measure unit 2400 and a KonicaMinolta CS200 luminance meter, respectively. External quantum efficiencies were calculated from front luminances, current densities and EL spectra.

## Electronic supplementary material

Supplementary Information
