# Supplementary material for: Dual mode OPV-OLED device with photovoltaic and light-emitting functionalities
Source: Sci Rep. 2018 Jul 31;8:11472. doi: 10.1038/s41598-018-29806-8 (PMC6068190; doi:10.1038/s41598-018-29806-8)
Supplement: Supplementary file 1 — Supplementary Information [file 41598_2018_29806_MOESM1_ESM.docx]

**Supplementary Information**

**Dual mode OPV-OLED device with photovoltaic and light-emitting functionalities**

*Takayuki Chiba,^*^ Daichi Kumagai, Kazuo Udagawa, Yuichiro Watanabe and Junji Kido^*^*

Graduate School of Organic Materials Science, Yamagata University,

4-3-16 Jonan, Yonezawa, Yamagata 992-8510, Japan

**
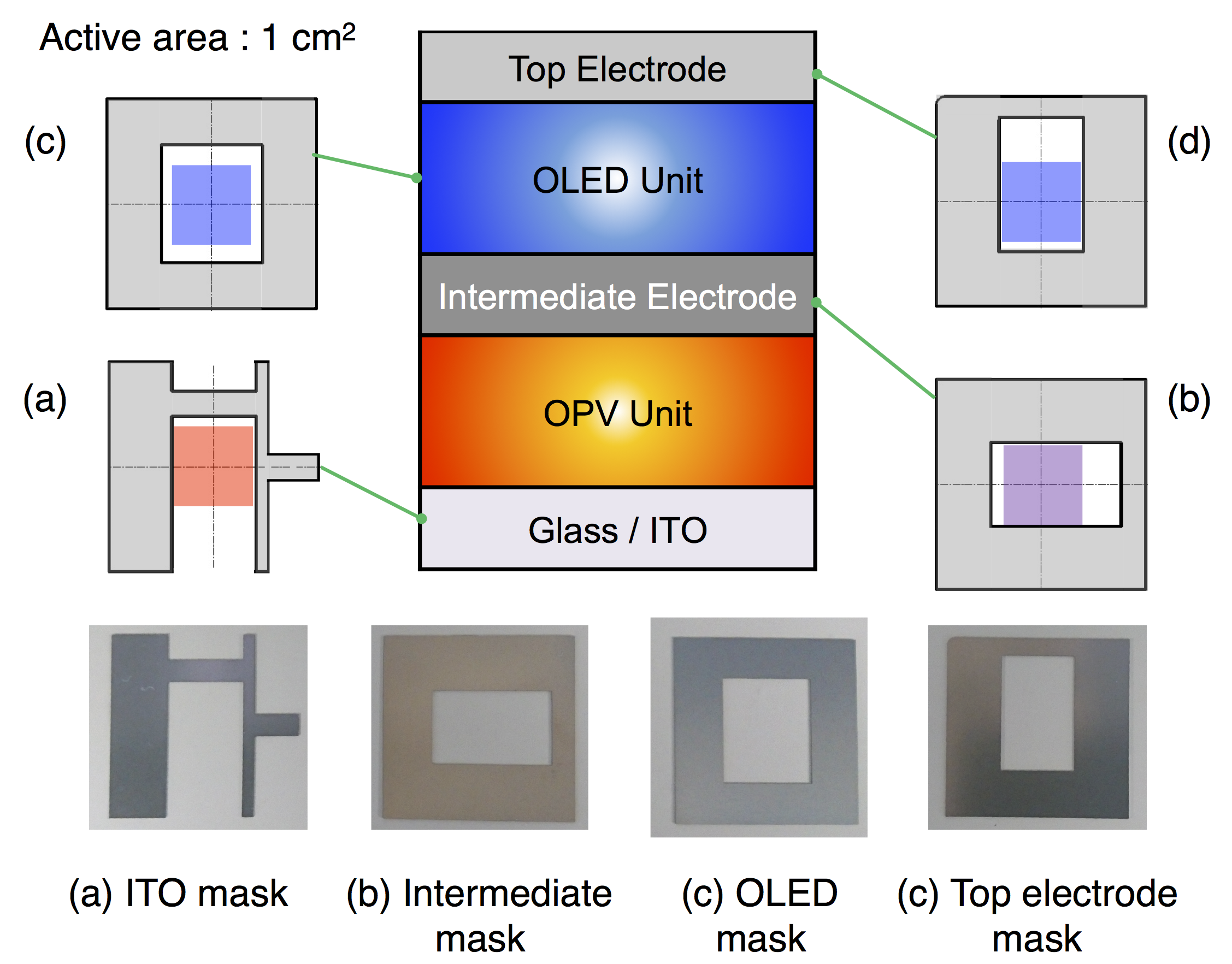
**

**Figure S1**. The deposition masks of the dual mode OPV-OLED device.


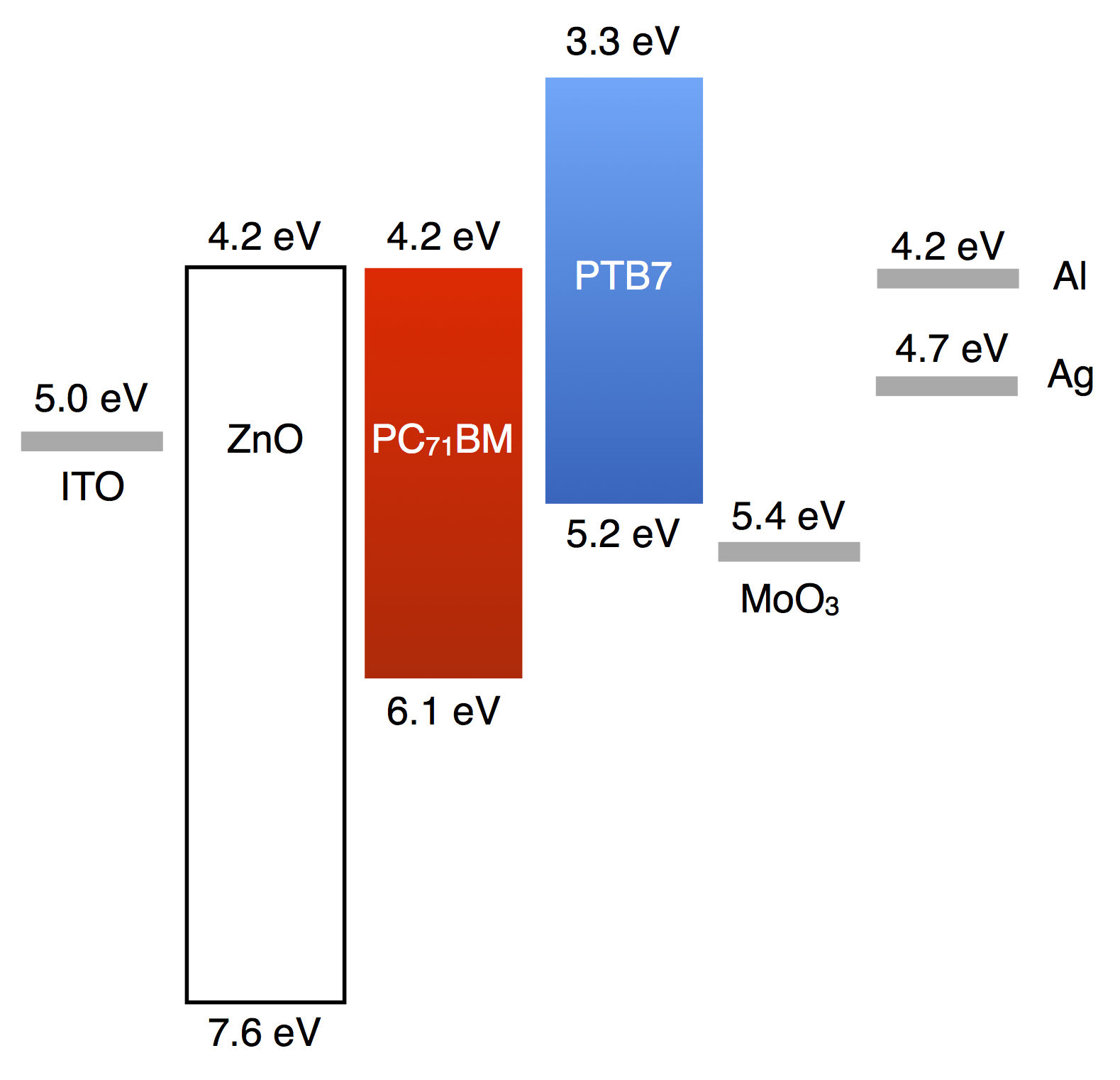


**Figure S2**. Energy diagrams of the polymer-based bulk-heterojunction inverted-OPV unit.

**Figure S3**. Transmittance of annealed and non-annealed sputter-deposited ITO films without and with annealing process.


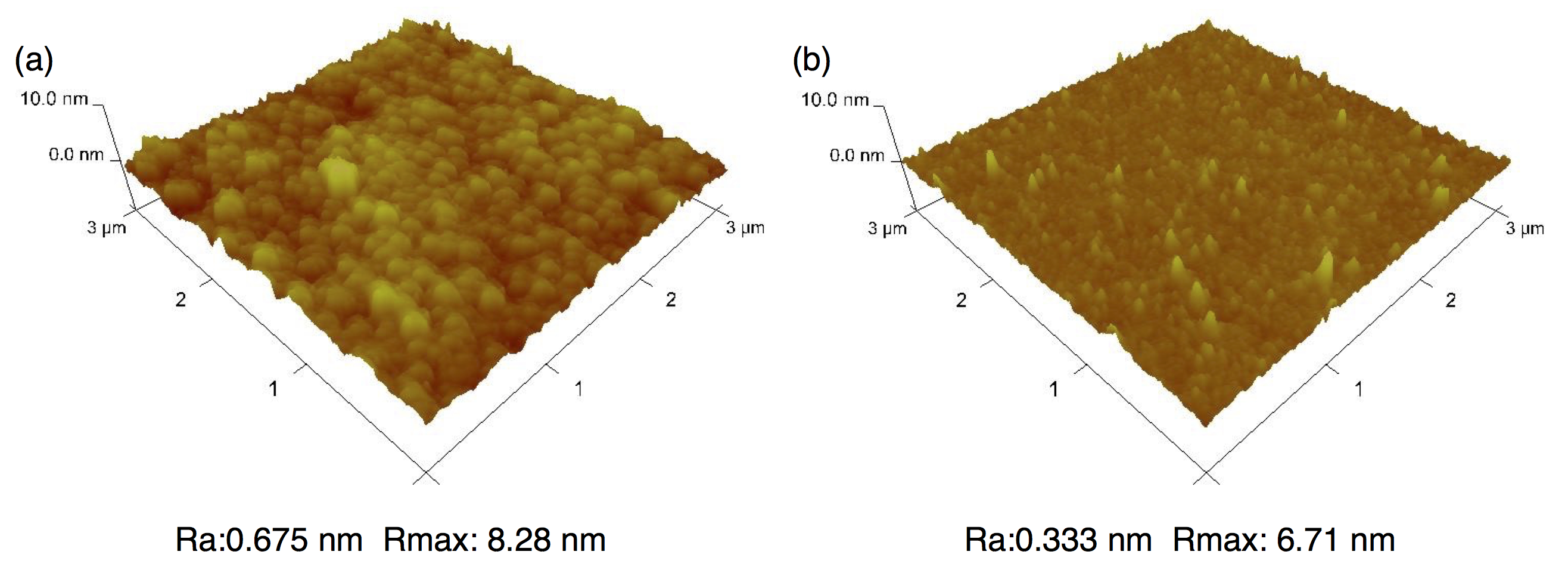


**Figure S4**. AFM images of (a) non-annealed and (b) annealed at 200 ˚C ITO films.

**Figure S5**. UV–vis absorption spectrum of PTB7:PC71BM film (2:3 by w/w).


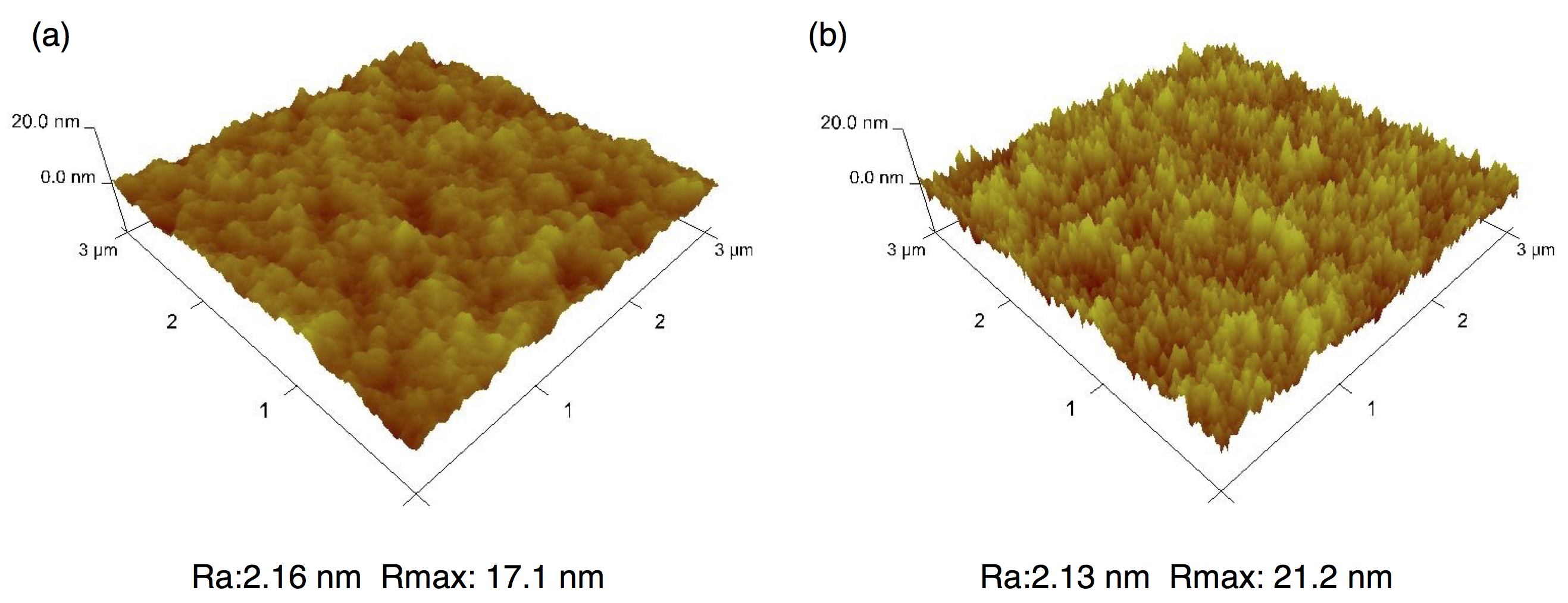


**Figure S6**. AFM images of (a) inverted OPV unit of ITO/ZnO/PTB7:PC_71_BM, (b) 60 wt% Ag-doped Al film deposited onto ITO/ZnO/PTB7:PC_71_BM/MoO3/Ag.

**Figure S7**. Energy diagram of the top-emission white OLED.

**Figure S8**. EL spectra of the (a) dual mode OPV-OLED and (b) single OLED.

**Figure S9**. *J-V* curves (OLED) of the dual mode OPV-OLED with a Ag/Al intermediate anode.

**Figure S10**. Single OLEDs characteristics. (a) *J-V-L* and (b) EQE-L curves of device with Ag-doped Al electrode. (c) *J-V-L* and (d) EQE curves of device with non-doped Al electrode.
